# Reviewing without a Clinical Background Is Detrimental for Cancer Pain Management

**DOI:** 10.3390/cancers11071005

**Published:** 2019-07-18

**Authors:** Sebastiano Mercadante

**Affiliations:** Supportive/Palliative Care Unit, La Maddalena Cancer Center, Via San Lorenzo 312, 90146 Palermo, Italy; terapiadeldolore@lamaddalenanet.it

## Abstract

Reviews are a fundamental space for summarizing and spreading knowledge on a particular topic. Methodologic skills may improve the clarity and the meaning of data presentation. A recent editorial choice provided an advanced update on a topic such as cancer pain, providing meaningful and appropriate information on hot topics of cancer pain management. Recent reviews have reported strange and misleading data, suggesting to some adjuvant drugs or opioids for mild-moderate pain instead of opioids on the basis of an incomprehensible analysis performed without any clinical sense. This is a serious problem because such information, published in an authoritative journal, could dis-educate oncologists in their daily practice.

A recent editorial choice recently provided an update by experts in the field to offer an overview of cancer pain management in some critical different aspects, also preparing a special and successful issue from different authors entitled “Cancer Pains” [1,2,3,4]. This decision is worthy, as it addresses common problems encountered by oncologists in their daily practice, for which an expert advice may be required.

In a world in which reviews of previous reviews are increasingly published, looking for misleading evidence and leading scientists to rise up against statistical significance [5], a pragmatic approach may better select data reflecting the clinical reality, instead of indicating unlikely clinical evidence [6]. I was dismayed and shocked when reading the results of a recent review [7], in part because it was published in an authoritative journal. It is suggested that non-opioid analgesics and NSAIDs can be effective as opioid therapy, in contrast to the presumed superiority of opioids. On the basis of the value of a meta-analysis in terms of evidence, these indications should be strictly followed by clinicians. Thus, we were informed that lidocaine, codeine plus aspirin, and pregabalin are the most effective treatments for cancer pain. They also added that, based on these promising findings, transdermal and subcutaneous formulations of lidocaine could be an important option. Furthermore, the combination of codeine plus aspirin was superior to strong opioids and this category of drugs might not even be necessary. In this rank order, there was pregabalin (they pointed out that there is no opioid constituent in its formulation). The evidence of these studies supported the conclusion that opioids are not necessary for chronic cancer pain. This statement was based on integrating direct and indirect comparisons in a model enabling formal comparisons between various classes and individual therapies. In the authors’ opinion, this work is relevant to clinical practitioners, because it reports explicit, quantitative comparisons between various drug classes and individual interventions for chronic cancer pain. This is a Copernican revolution for researchers and clinicians, a sort of fantastic world where apples are mixed with pears, carrots, and so on with great ease. The reason for such misleading information is that it simply relies on a wrong acquisition and interpretation of data without the necessary knowledge of the drugs in question, their relative potency, the different context in which they are administered, and above all, without the minimal clinical sense. This is evident by the capacity to include among these categories, ziconotide, a drug that is used intrathecally [8]. Indeed, it is omitted that patients were receiving a median oral morphine equivalent dosage of 300–600 mg/d in 92% of patients, antidepressants (44%), anxiolytics (40%), anticonvulsants (27%), anti-inflammatory agents (28%), and antipsychotics (16%). On the other hand, lidocaine has been recently examined in a more equilibrated review. It was administered as an infusion. There were one positive and three negative trials evaluating lidocaine versus placebo for short periods [9]. Most of these studies were performed in patients who were receiving opioids. How can we say that lidocaine works universally while opioids not?

Pregabalin has been also reported as an effective drug. One study was stopped for slow recruitment and, again, it was omitted that stable and optimized opioid treatment was an entry criterion for the study [10]. In another one, a multiple combination of drugs were compared, including amitryptiline, in patients on opioids [11]. In another study, pregabalin and transdermal fentanyl were compared in patients with neuropathic pain. This study provided unrealistic data reporting doses of pregabalin titrated up to 600 mg/day associated with an acceptable profile of adverse effects. This disproportionate data, unobservable in a clinical context, arouses serious doubts, as the principal investigator was then employed at the pharmaceutical company of the product [12]. Is there a researcher that can reproduce data suggesting that pregabalin works as a sole analgesic in cancer patients, even with a neuropathic component? For instance, pregabalin added to morphine did not provide any substantial analgesic benefit [13]. More meaningful reviews regarding antiepileptic drugs, do not support such hypothesis, even when they are given on top of opioid therapy [14].

Can we really assert that codeine-aspirin combination would be the solution for cancer pain management, setting aside strong opioids? Should studies of one or few days in duration, other than being of bad quality, suggest clinicians to consider this drug or this combination a solution that may avoid the use of more potent opioids? (By the way, codeine is pro-drug needing to be transformed to morphine to provide an analgesic effect…) [15]. In which stage and circumstances were the patients included? Can we seriously say that this combination could be given for months successfully without losing efficacy, if any initially, when the disease progresses? Or do they mean that patients who receive opioids for very difficult pain syndromes could be switched to codeine, improving their condition? For instance, about 19 out of 20 cancer patients with moderate or severe pain who are given opioids will have their pain reduced to mild or no pain within 14 days [16].

Of interest, the overwhelming and unjust criticism of opioid analgesics in cancer pain management coincides with a growing media and marketing activity of non-opioid alternatives (e.g., cannabinoids). There are a plethora of “systematic reviews” and “reviews of systematic reviews” regarding cannabinoids, and that problem has also been raised lately in a recent article, where it is pointed out that the number of “systematic reviews” on cannabinoids exceeds couple-fold the number of the low-quality RCTs [17].

What is worrying is that oncologists, for whom a respectable journal represents a reliable source of data, can therefore take these fanciful interpretations literally and make them their own in clinical practice. This would mean that cancer pain could be treated by the category of oncologists with lidocaine, codeine and non-opioids, and pregabalin, without the need of opioids. If I were to read this information on a cockeyed website in internet, I would say that this is fake news and move on. However, this misleading information is available to oncologists or doctors dealing with cancer patients from a scientific source. They should disregard and critically consider aggregated studies possibly performed by researchers without adequate experience in cancer pain management. Pain is often neglected by the oncologist. Further training and awareness may result in better cancer pain management and better qualifying misleading information from literature reviews.
